# Presidential address 2025: expansion of computer-based testing from 12 to 27 health professions by 2027 and adoption of a large language model for item generation

**DOI:** 10.3352/jeehp.2025.22.7

**Published:** 2025-01-20

**Authors:** Hyunjoo Pai

**Affiliations:** President, Korea Health Personnel Licensing Examination Institute, Seoul, Korea; Hallym University, Korea

The year 2025 has arrived. First, I hope that this year will bring health and happiness to all individuals visiting the *Journal of Educational Evaluation for Health Professions* (JEEHP) as we celebrate this promising new year. I also express my deepest gratitude to those who have diligently contributed to research and education within the healthcare field. Your dedication has allowed JEEHP to continue growing and fulfilling its mission of advancing healthcare education and evaluation.

## Achievements of the journal

In 2024, JEEHP attained significant milestones. As reported by Clarivate Analytics, the journal achieved a Journal Impact Factor (JIF) of 9.3, positioning it as the leading journal among 85 SCIE/ESCI-indexed journals worldwide in the field of science education [1]. Furthermore, JEEHP secured the top spot (10.19) among Korean academic journals in the Cite/Doc (2 years) indicator as published by SCImago Journal & Country Rank. These accomplishments were made possible through the contributions of many, including the editor-in-chief and the editorial board members. I extend my heartfelt appreciation to all those who have committed themselves to the advancement of JEEHP.

## Expansion of computer-based testing

The Korean Health Personnel Licensing Examination Institute (KHPLEI) is undergoing a significant transformation and facing new challenges with the expansion of computer-based testing (CBT) in 2025. Currently, CBT is being utilized for 12 national licensing examinations, including those for physicians, dentists, oriental medical doctors, and care workers [2]. We plan to extend the adoption of CBT to various other national licensing examinations for healthcare professionals. Starting in 2025, CBT will also be introduced for the licensing examinations of nurse assistants, optometrists, and speech-language pathologists.

As a result, by 2027, 24 out of the 34 national licensing examinations will be conducted as CBT. This format not only enhances the quality of clinical-centered assessment by diversifying question types—such as through the use of multimedia—but also improves the convenience for test-takers by reducing the time spent on problem-solving and answer-writing while facilitating easier reviews and corrections.

## Adoption of generative artificial intelligence for item generation

The KHPLEI will continue to strive to expand CBT and develop diverse types of items suitable for the CBT environment, enabling proactive adaptation to the rapidly evolving medical landscape. Furthermore, we will conduct research on developing test items using generative artificial intelligence, including large language models, which are rapidly progressing. JEEHP will remain committed to sharing innovative and practical research findings in educational evaluation within the healthcare field and to providing a platform for diverse academic discussions that align with these trends.

We look forward to providing even richer content in our journal throughout 2025, with the continued participation and engagement of researchers in the healthcare field. We kindly request your continued interest and feedback to support the development of JEEHP, and we remain open to your valuable suggestions at all times.

Lastly, we would like to once again express our sincere gratitude to our editor and editorial board members for their unwavering dedication to JEEHP. We also wish all our authors and readers good health and happiness in the year ahead. Thank you.
